# Aluminum tolerance association mapping in triticale

**DOI:** 10.1186/1471-2164-13-67

**Published:** 2012-02-13

**Authors:** Agnieszka Niedziela, Piotr T Bednarek, Henryk Cichy, Grzegorz Budzianowski, Andrzej Kilian, Andrzej Anioł

**Affiliations:** 1 Plant Breeding and Acclimatization Institute, 05-870 Błonie, Radzików, Poland; 2 Plant Breeding Company-Strzelce, Breeding Department Małyszyn, 66-400 Gorzów Wlkp., Myśliborska Str. 81, Poland; 3 Diversity Array Technology Pty Ltd, 1 Wilf Crane Crescent, Yarralumla, ACT 2600, Australia

## Abstract

**Background:**

Crop production practices and industrialization processes result in increasing acidification of arable soils. At lower pH levels (below 5.0), aluminum (Al) remains in a cationic form that is toxic to plants, reducing growth and yield. The effect of aluminum on agronomic performance is particularly important in cereals like wheat, which has promoted the development of programs directed towards selection of tolerant forms. Even in intermediately tolerant cereals (i.e., triticale), the decrease in yield may be significant. In triticale, Al tolerance seems to be influenced by both wheat and rye genomes. However, little is known about the precise chromosomal location of tolerance-related genes, and whether wheat or rye genomes are crucial for the expression of that trait in the hybrid.

**Results:**

A mapping population consisting of 232 advanced breeding triticale forms was developed and phenotyped for Al tolerance using physiological tests. AFLP, SSR and DArT marker platforms were applied to obtain a sufficiently large set of molecular markers (over 3000). Associations between the markers and the trait were tested using General (GLM) and Multiple (MLM) Linear Models, as well as the Statistical Machine Learning (SML) approach. The chromosomal locations of candidate markers were verified based on known assignments of SSRs and DArTs or by using genetic maps of rye and triticale.

Two candidate markers on chromosome 3R and 9, 15 and 11 on chromosomes 4R, 6R and 7R, respectively, were identified. The *r^2 ^*values were between 0.066 and 0.220 in most cases, indicating a good fit of the data, with better results obtained with the GML than the MLM approach. Several QTLs on rye chromosomes appeared to be involved in the phenotypic expression of the trait, suggesting that rye genome factors are predominantly responsible for Al tolerance in triticale.

**Conclusions:**

The Diversity Arrays Technology was applied successfully to association mapping studies performed on triticale breeding forms. Statistical approaches allowed the identification of numerous markers associated with Al tolerance. Available rye and triticale genetic maps suggested the putative location of the markers and demonstrated that they formed several linked groups assigned to distinct chromosomes (3R, 4R, 6R and 7R). Markers associated with genomic regions under positive selection were identified and indirectly mapped in the vicinity of the Al-tolerant markers. The present findings were in agreement with prior reports.

## Background

Hexaploid triticale (X Triticosecale Wittmack) is a hybrid of tetraploid wheat and diploid rye with genome composition AA, BB and RR. It is cultivated in Poland mainly as a fodder cereal, and its area of cultivation doubled during the last 10 years [1]. Triticale is frequently grown on acid soils in the presence of excessive toxic aluminum ions that inhibit root growth and seed yields [2]. The development of tolerant cultivars, including triticale, is an important breeding objective.

Tolerant plants can be identified by physiological tests [3-9]. This robust approach is inexpensive and generally provides a reliable measure of tolerance [4]. It relies on morphological traits that may not be directly related to the expression of Al-tolerant genes in response to environmental factors. More direct methods based on DNA markers [10-13] and saturated genetic maps or, preferentially, consensus maps of the species are needed to overcome these issues.

One of the most promising marker platforms for such studies is Diversity Arrays Technology (DArT) [14], which enables the identification of thousands of highly reliable markers in a single run [15-17]. DArT performs well in many species in which several markers can be assigned to individual chromosomes [15,18]. This system is also available for triticale and the first genetic map saturated with DArTs was recently published [19]. Moreover, AFLPs and SSR markers can be used for such studies [20-22].

There are at least two approaches to the identification of markers useful for marker-assisted selection (MAS). The first one is based on biparental mapping populations and it allows the identification of QTLs and linked markers [23,24], while the second method involves the study of "non-Mendelian" populations and is called association mapping [25,26]. The "Mendelian" approach enables only the identification of loci segregating in tested samples of a given cross. Thus, many different mapping populations may be needed to represent the allelic diversity of the genes contributing to the character under study in the tested species. Moreover, alleles present in some crosses may not always be expressed with major effects in other crosses within the species or may not be present there at all [27,28]. In contrast, the association mapping approach allows for the identification of many loci and alleles among all the individuals of the population [26]. This type of analysis was done in maize, lettuce, barley, wheat, oat and soybean [29-36]. However, in the absence of genetic maps, markers obtained through association mapping cannot be assigned to the respective chromosomes.

Additional sets of markers useful for MAS could be derived from markers representing genomic regions under putative selection pressure [37]. Genomic regions under positive selection pressure may be bound to adaptation traits in breeding materials [38,39]. Such markers would be of special value if mapped in the region of known QTLs.

The initial attempts to identify the chromosomal location of the Al-tolerant QTLs were performed on the wheat-triticale substitution lines [40-42]. The rye chromosomes number 3, 4, and 6 seem to contain major Al tolerance-related genes [40-42]. Aluminum tolerance also appeared to be controlled by certain wheat genes, but their location on chromosomes in triticale is unknown [43]. Little is known about the genes coding for this trait in triticale [41]. Nevertheless, studies performed in several octoploid triticale genotypes demonstrated that Al tolerance was associated with a high level of citrate exudation from roots [43], which is mediated by a transporter encoded by a Multidrug and Toxin Efflux (MATE) family gene mapped to 4BL in wheat [11]. In addition, studies in wheat suggested that Aluminum-Activated Malate Transporter (ALMT) family genes located on 4DL may participate in the trait [44]. A gene belonging to the same family was also identified in rye on the 7RS chromosome [45]. DNA markers based on the sequences of such genes may be used by breeders for MAS in triticale, although the causal link between these genes and Al resistance remains to be established.

The present study aimed to identify molecular markers associated with Al tolerance among plants randomly selected from advanced triticale breeding materials. The association mapping approach was applied, using General (GLM) and Multiple Linear (MLM) Models, as well as Statistical Machine Learning (SML). The chromosomal location of the markers was determined based on available genetic maps of triticale and rye.

## Results

### Al tolerance test

Out of 232 individual plants representing 232 breeding forms, the roots of 76 plants were irreversibly damaged by aluminum and did not continue to grow after the test, and 35 showed little regrowth ability (transformed value of regrowth below 0.2 (Table 1)). Both types of individuals were classified as non-tolerant (N). The plants with transformed regrowth between 0.2 and 0.5 were called moderately tolerant (I), while those with regrowth greater than 0.5 were classified as tolerant (T). Among the individuals representing breeding forms of triticale, only three were more tolerant than rye cv. Dańkowskie Złote and just one exceeded cv. Strzekęcińskie, which were used as controls (not shown).

**Table 1:** Number of spring and winter triticale individuals representing 232 breeding forms classified as Al-tolerant, moderately tolerant, and non-tolerant groups based on arcsine transformed values of regrowth

Triticale forms	Classification		
	
	Non-tolerant	Moderately tolerant	Tolerant
	
	< 0.20	0.21-0.50	> 0.50
Spring	1	17	21

Winter	110	53	30

Total	111	70	51

### Genotyping

The marker platforms used allowed the identification of 3289 polymorphic markers: 3117 DArT, 145 AFLP and 27 SSR. After the removal of redundant markers, the number was reduced by nearly one half, as shown for certain chromosomes of rye in Table 2.

**Table 2:** Number of molecular markers assigned to 3R, 4R, 6R and 7R chromosomes according to marker type and divided into redundant and non-redundant classes

Marker type	Redundant (R)/Non-redundant (NR)	Chromosome			
		
		3R	4R	6R	7R
DArT	R	68	209	186	124
	
	NR	38	147	117	77

SSR	R	-	-	-	93
	
	NR	-	-	-	48

### Agglomeration analysis

Clustering of the individuals in the 3R set resulted in the formation of two groups (100% of bootstrapping, not shown). Similar analyses of the 4R and 7R sets also revealed the presence of two groups with high bootstrap value. However, no structuring was present in the 6R marker set. In all cases, the groups did not correspond to the Al tolerance trait shown in the physiological test.

### Structure identification

The *ad hoc *statistic ΔK revealed strong data structuring for the 3R, 4R and 7R marker sets with K equal to 2. The 6R set exhibited weak structuring with two putative groups of individuals. In contrast to the agglomeration analyses, Bayesian statistics grouped the sets according to aluminum tolerant phenotypes. The moderately tolerant forms were grouped with the tolerant ones. The number of individuals classified into a given group varied from chromosome to chromosome (Table 3), possibly due to the difference in the number of individuals present after merging procedures in each chromosomal set.

**Table 3:** Arrangement of Al tolerance associated markers (AS), redundant markers and outliers under positive (PS) and balancing (BS) selection for separate chromosomes and they localization on available genetic maps of triticale [19] and rye [15]

Chromo-some	Structure ΔK (extreme value)	Associated marker (AS)/outlier PS and BS	Association mapping									Markers at the same position on the available maps as the associated, redundant and markers under selection	Rye map [15] (cM)	**Triticale map **[19] (cM)
						
			TASSEL							SML				
						
			Marker name	Redundant markers	GML (*p*-value)	***r***^**2**^	MLM (*p*-value)	***r***^**2**^	Bonferroni test	Marker name	*p-*value (whiteout assuming data structure)			
3R	K2 (3 100)	AS	**wPt-3564**	-	3.63E-09	0.220	7.92E-04	0.117	1.31E-03	**wPt-3564**	0.0000	-	177.6	-
					
			-	-	-	-	-	-		rPt-401520	0.0145	rPt-507396, rPt-507318	308.4	-
		
		PS	rPt-400318	-	-	-	-	-	-	-	-	-	-	-
			
			rPt-508819	-	-	-	-	-	-	-	-	-	-	-
			
			rPt-390252	-	-	-	-	-	-	-	-	rPt-400789	349.3	-
			
			rPt-508975	-	-	-	-	-	-	-	-	rPt-400864, rPt-402392, rPt-400152, wPt-6762, wPt-7540	-	50.6
			
			rPt-2965	-	-	-	-	-	-	-	-	rPt-346892, rPt-347072, rPt-346946, rPt-347454, rPt-346755	109.4	-
			
			rPt-402334	-	-	-	-	-	-	-	-	-	-	-

4R	K2 (8 300)	AS	**rPt-505674**	-	1.99E-07	0.146	8.01E-07	0.153	3.40E-04	**rPt-505674**	0.0194	rPt-507649, rPt-401692	160.1	-
					
			rPt-507784	-	8.02E-07	0.152	4.23E-05	0.126		-	-	rPt-507581	348.4	-
					
			**rPt-401376**	-	1.74E-06	0.144	1.53E-04	0.107		**rPt-401376**	0.0125	rPt-390125, rPt-346609,	163.8	-
						
				rPt-399885	-	-	-	-		-	-	rPt-509441, rPt-513924, rPt-508199, rPt-1422, rPt-400270, rPt-6513, rPt-399885, tPt-5795	160.7	67.3
						
				rPt-390125	-	-	-	-		-	-	-	163.8	-
						
				rPt-400270	-	-	-	-		-	-	-	161.3	67.3
						
				rPt-389881	-	-	-	-		-	-	-	163.2	-
					
			rPt-402237	-	4.07E-06	0.135	5.22E-06	0.152		-	-	rPt-401410	124.3	-
					
			rPt-508577	-	2.30E-05	0.099	1.36E-04	0.088		-	-	-	-	-
					
			rPt-411417	-	3.71E-05	0.094	1.65E-04	0.086		-	-	-	7.1	
					
			**rPt-402563**	-	3.79E-04	0.088	3.49E-03	0.068		**rPt-402563**	0.0087	-	-	-
			
			-	-	-	-	-	-	-	rPt-509188	0.0135	rPt-402590, rPt-389763	94.0	-
			
			-	-	-	-	-	-	-	rPt-410768	0.0184	rPt-507981	238.1	-
			
		PS	rPt-505775	-	-	-	-	-	-	-	-	-	-	-
			
			rPt-505352	-	-	-	-	-	-	-	-	rPt-402302, rPt-506593, rPt-398512 rPt-389700, rPt-9611, rPt-509695, rPt-7906, rPt-400317, rPt-401825, tPt-514203, rPt-507403, tPt-512937, rPt-505288, wPt-9994	154.3	58.4
			
			rPt-400317	-	-	-	-	-	-	-	-	rPt-389700, rPt-9611, rPt-509695, rPt-7906, rPt-505775, rPt-401825, tPt-514203, rPt-507403, tPt-512937, rPt-505288, wPt-9994	153.7	58.4
		
		BS	rPt-507894	-	-	-	-	-	-	-	-	rPt-506357, rPt-399506, rPt-509554, rPt-509722, tPt-512921, wPt-2714	139.1	55.1
			
			rPt-506357	-	-	-	-	-	-	-	-	rPt-507894	139.1	-
			
			rPt-506527	-	-	-	-	-	-	-	-	-	-	-
			
			rPt-411417	-	-	-	-	-	-	-	-	-	7.1	-
			
			rPt-389465	-	-	-	-	-	-	-	-	-	-	-
			
			rPt-508454	-	-	-	-	-	-	-	-	-	-	-
			
			rPt-389455	-	-	-	-	-	-	-	-	-	-	-
			
			rPt-506540	-	-	-	-	-	-	-	-	-	-	-
			
			rPt-507403	-	-	-	-	-	-	-	-	tPt-512937	-	59.5
			
			rPt-508638	-	-	-	-	-	-	-	-	-	-	-
			
			rPt-508722	-	-	-	-	-	-	-	-	rPt-507894, rPt-399506, rPt-509554, tPt-512921, wPt-2714	-	55.1
			
			rPt-506534	-	-	-	-	-	-	-	-	wPt-8954	-	52.9
			
			rPt-402302	-	-	-	-	-	-	-	-	rPt-506593, rPt-505352, rPt-398512	154.0	-

6R	K2 (88)	AS	rPt-399834	-	3.75E-09	0.178	1.67E-07	0.169		-	-	-	-	-
						
				rPt-507199	-	-	-	-		-	-	rPt-8205	-	43.4
						
				rPt-507896	-	-	-	-		-	-	rPt-3995874, rPt-399777, rPt-399399, rPt-506885, tPt-513728, rPt-507896, rPt-399587, rPt-411022, rPt-401093, rPt-390525	-	45.6
					
			rPt-402561	-	3.32E-08	0.158	8.42E-07	0.150	4.27E-04	-	-	wPt-6978, wPt-2013, wPt-1320, rPt-506799, rPt-505233, rPt-400005, rPt-400819	-	44.5
					
			rPt-390636	-	1.29E-06	0.126	8.13E-06	0.124		-	-	-	549.5	-
					
			rPt-401083	-	8.22E-06	0.110	2.96E-05	0.110		-	-	-	-	-
					
			rPt-402018	-	1.14E-05	0.107	8.97E-05	0.097		-	-	-	486.1	-
					
			rPt-402447	-	1.14E-05	0.107	8.97E-05	0.097		-	-	-	486.1	-
					
			rPt-507674	-	1.14E-05	0.107	8.97E-05	0.097		-	-	-	430.4	-
					
			rPt-402015	-	1.14E-05	0.107	8.97E-05	0.097		-	-	-	486.1	-
					
			rPt-508379	-	1.53E-05	0.105	5.14E-05	0.103		-	-	-	-	-
					
			rPt-399406	-	3.28E-05	0.098	1.73E-04	0.090		-	-	-	486.1	-
					
			rPt-505870	-	8.60E-05	0.089	2.26E-04	0.087		-	-	rPt-509502	540.6	85.7
			
			-	-	-	-	-	-	-	rPt-390698	0.016	rPt-389306, rPt-402575, wPt-1676, rPt-508661, tPt-6200, rPt-507438, rPt-401326, rPt-398845, wPt-8548 rPt-505525, wPt-2077, rPt-389414, rPt-508426, rPt-509027, rPt-401648, Pt-410992, rPt-400907, rPt-389714, tPt-512866, Xrems1247	93.0	4.4
			
			-	-	-	-	-	-	-	rPt-509167	0.006	-	556.9	-
			
			-	-	-	-	-	-	-	rPt-506198	0.007	-	-	-
			
			-	-	-	-	-	-	-	rPt-505347	0.011	-	-	-
		
		PS	rPt-401893	-	-	-	-	-	-	-	-	rPt-389665, rPt-508711	148.6	-
		
		BS	rPt-401470	-	-	-	-	-	-	-	-	-	-	-
			
			rPt-389991	-	-	-	-	-	-	-	-	-	-	-
			
			rPt-411086	-	-	-	-	-	-	-	-	-	-	-
			
			rPt-505673	-	-	-	-	-	-	-	-	-	-	-
			
			rPt-506099	-	-	-	-	-	-	-	-	rPt-509333, rPt-509333, rPt-506902, rPt-399825, rPt-411161, rPt-399879, wPt-6868, wPt-0935	381.9	51.2
			
			rPt-509333	-	-	-	-	-	-	-	-	-	-	-
			
			rPt-402561	-	-	-	-	-	-	-	-	wPt-6978, wPt-2013, wPt-1320, rPt-506799, rPt-505233, rPt-400005, rPt-400819	-	44.5
			
			rPt-399991	-	-	-	-	-	-	-	-	-	-	-
			
			rPt-399245	-	-	-	-	-	-	-	-	-	-	-
			
			rPt-509728	-	-	-	-	-	-	-	-	-	-	-
			
			rPt-390337	-	-	-	-	-	-	-	-	-	-	-
			
			rPt-400060	-	-	-	-	-	-	-	-	-	-	-
			
			rPt-508690	-	-	-	-	-	-	-	-	-	-	-
			
			rPt-401554	-	-	-	-	-	-	-	-	-	-	-

7R	K2 (15 300)	AS	rPt-401366	-	1.83E-08	0.177	1.09E-05	0.130		-	-	-	-	-
						
				rPt-506317	-	-	-			-	-	-	-	-
						
				rPt-508078	-	-	-			-	-	-	-	-
						
				rPt-509357	-	-	-			-	-	-	-	-
					
			rPt-509359	-	1.83E-08	0.177	1.09E-05	0.130		-	-	-	-	-
					
			**rPt-505798**	-	1.83E-08	0.177	1.09E-05	0.130		**rPt-505798**	0.0096	-	-	-
					
			**rPt-508078**	-	1.83E-08	0.177	1.09E-05	0.130		**rPt-508078**	0.0009	-	-	-
					
			rPt-509056	-	6.03E-08	0.186	4.23 E-06	0.169		-	-	-	232.8	-
					
			rPt-505154	-	6.03E-08	0.186	4.23 E-06	0.169	2.00E-03	-	-	-	232.8	-
					
			**rPt-8750**	-	3.61E-06	0.144	3.25 E-05	0.139		**rPt-8750**	0.0038	-	-	-
					
				rPt-399570	-	-	-	-		-	-	-	-	-
					
				rPt-401526	-	-	-	-		-	-	rPt-398753, rPt-400372, wPt-2793, rPt-400061, wPt-345783, rPt-506142, wPt-4738, rPt-506028, wPt-11703, rPt-508380	-	35.7
							
				rPt-400816	-	-	-	-		-	-		-	
							
				rPt-399664	-	-	-	-		-	-		-	
					
				rPt-399292	-	-	-	-		-	-	-	-	-
					
				rPt-390741	-	-	-	-		-	-	-	-	-
					
			SCM92_177	-	8.57E-06	0.135	9.05 E-04	0.092		-	-	-	-	-
					
			rPt-390593	-	1.85E-05	0.126	1.28 E-03	0.087		-	-	-	-	-
						
				rPt-401828	-	-	-	-		-	-	-	-	-
					
			tPt-8771	-	4.67E-05	0.116	3.70 E-04	0.104		-	-	-	-	-
					
			**rPt-399325**	-	8.95E-05	0.090	1.39 E-03	0.066		**rPt-399325**	0.005	-	-	92.7
		
		PS	rPt-400793	-	-	-	-	-	-	-	-	-	-	-
			
			SCM16_259	-	-	-	-	-	-	-	-	-	-	-
		
		BS	rPt-401363	-	-	-	-	-	-	-	-	-	-	-
			
			rPt-509288	-	-	-	-	-	-	-	-	-	-	-
			
			rPt-401221	-	-	-	-	-	-	-	-	-	-	-
			
			SCM92_295	-	-	-	-	-	-	-	-	-	-	-
			
			rPt-389372	-	-	-	-	-	-	-	-	-	-	-
			
			rPt-506250	-	-	-	-	-	-	-	-	-	-	-
			
			rPt-399665	-	-	-	-	-	-	-	-	-	-	-
			
			rPt-508868	-	-	-	-	-	-	-	-	-	-	-
			
			rPt-402262	-	-	-	-	-	-	-	-	-	-	-
			
			rPt-390750	-	-	-	-	-	-	-	-	-	-	-

Bolded markers - markers associated according to both methods (TASSEL and SML). ΔK indicates the most probable structure of the data. In bracket the extreme of the ΔK is indicated. Significance of the associated markers is indicated for GLM, MLM and SML. The *r*^2 ^and Bonferroni test *p*-values are also indicated for the reference.

### Association mapping

In the 3R chromosomal set, a single associated marker (wPt-3564) was identified by every approach (Table 3). Moreover, SML identified an additional significantly associated marker (rPt-401520). Analysis of the 4R set revealed seven associated markers identified by GLM and MLM analyses simultaneously. SML identified five associated markers, and three of them were common for all methods (Table 3). Among the 6R chromosome markers, eleven were associated with aluminum tolerance as indicated by GLM and MLM. The SML approach identified four additional markers that could be associated with the trait of interest. In the highly structured 7R set, eleven markers were associated with tolerance according to GML and MLM. Four of them were also detected by SML (Table 3). All of these markers passed the Bonferroni test (Table 3) and showed a good fit with the data (see *r^2 ^*parameter, Table 3) in most cases, with better results for the General Linear Model (GML) than for the Mixed Linear Model (MLM) approach.

The redundant counterparts of several associated markers were excluded from association mapping for the simplicity of the analyses and computation efficiency. This information is provided in Table 3.

### Positive and balancing selection

Among the 3R markers, six reflected genomic regions under putative positive selection pressure (Table 3). The 4R set was represented by three markers associated with positive selection, while thirteen were associated with balancing selection. Positive selection was also identified by a single marker and balancing selection by 14 markers in the 6R set. Finally, two markers of the 7R set were assigned to loci under positive and ten under balancing selection.

### Indirect mapping of Al-tolerant genes

**Figure 1:**
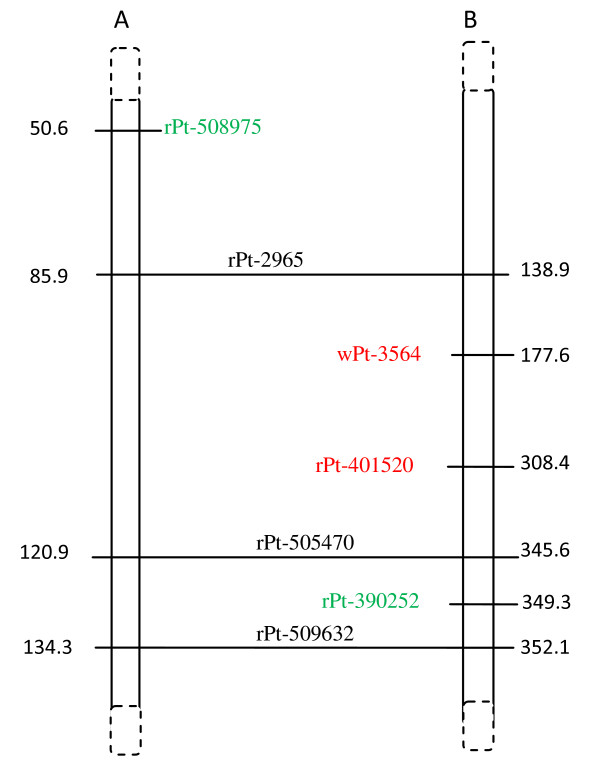
**Alignment of triticale (A) and rye (B) 3R genetic maps**. Associated and redundant markers (see also Table 3) are indicated in red. Markers in green correspond to those from genomic regions under putative positive selection, while those in blue are from regions under balancing selection. Markers common to both maps are shown in dark and are linked by horizontal lines. The distance is given in cM.

**Figure 2:**
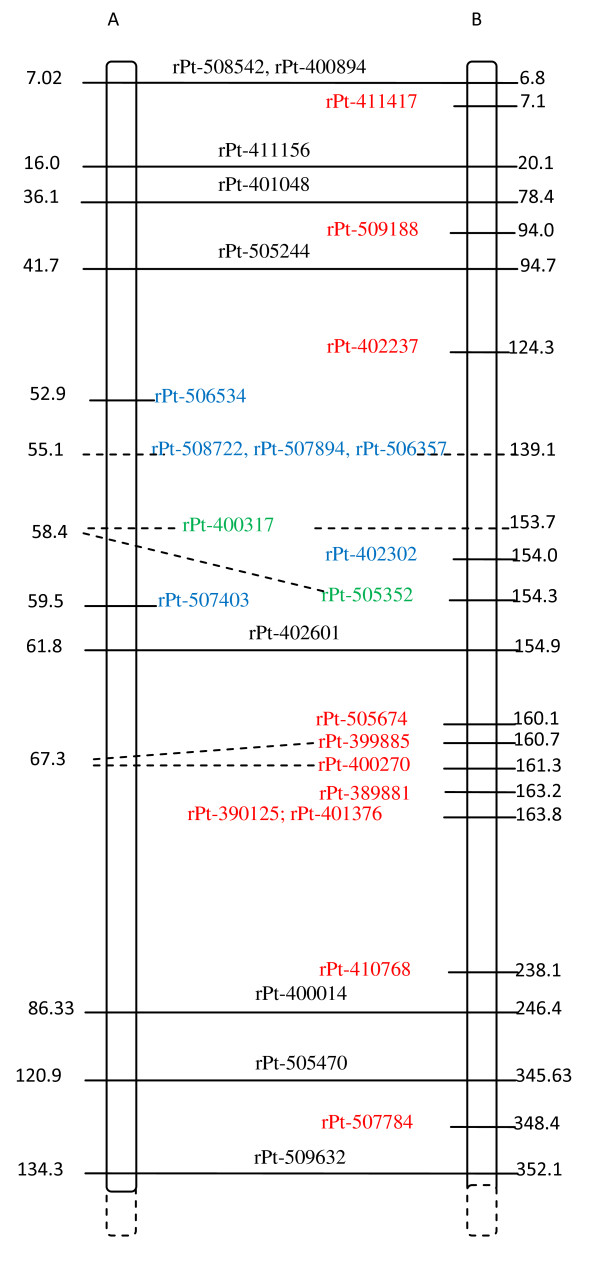
**Alignment of triticale (A) and rye (B) 4R genetic maps**. Associated and redundant markers (see also Table 3) are indicated in red. Markers in green correspond to those from genomic regions under putative positive selection, while those in blue are from regions under balancing selection. Markers common to both maps are shown in dark and are linked by horizontal lines. The distance is given in cM.

**Figure 3:**
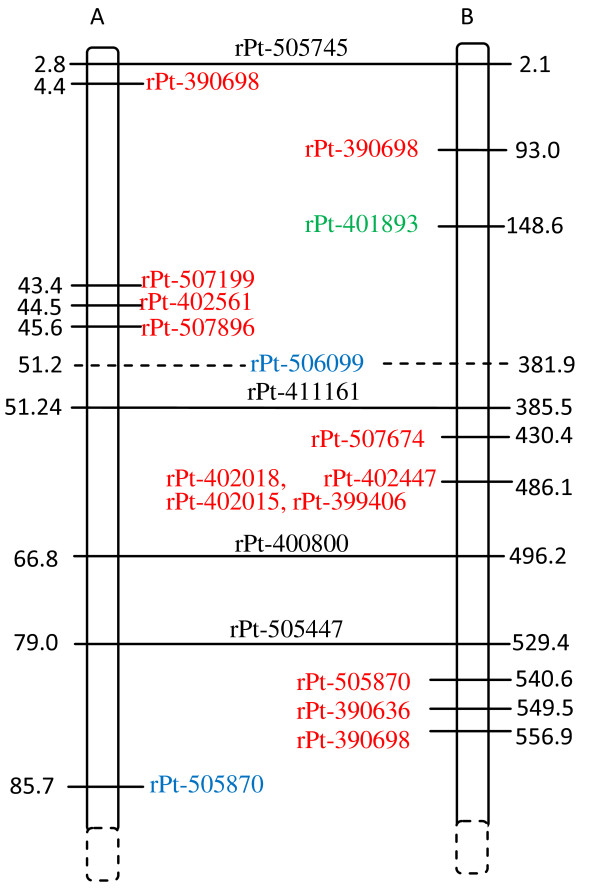
**Alignment of triticale (A) and rye (B) 6R genetic maps**. The associated markers and those belonging to the same redundant marker bin(s) (see also Table 3) are indicated in red. Markers in green correspond to genomic regions under putative positive selection, while those in blue are from regions under balancing selection. Markers common to both maps are shown in dark and are linked by horizontal lines. The distance is given in cM.

**Figure 4:**
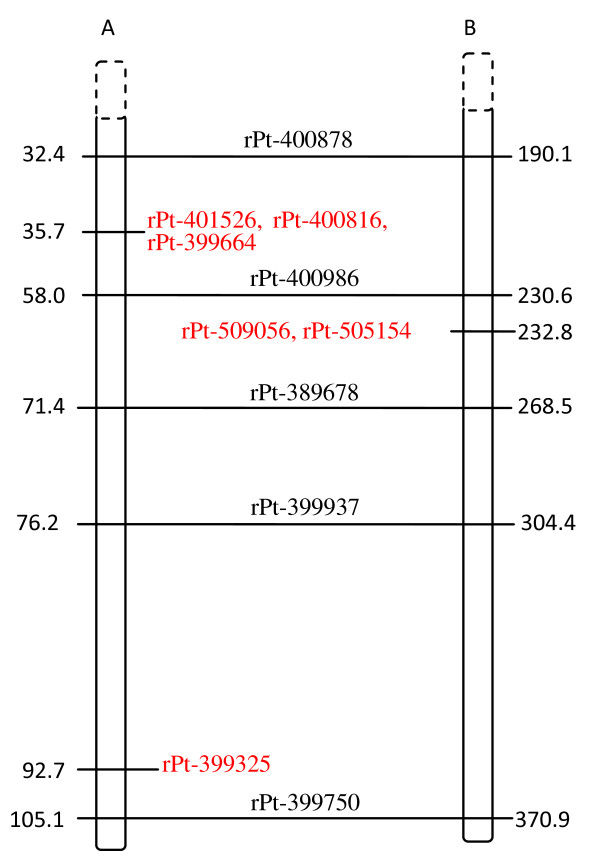
**Alignment of triticale (A) and rye (B) 7R genetic maps**. Associated and redundant markers (see also Table 3) are indicated in red. Markers common to both maps are shown in dark and are linked by horizontal lines. The distance is given in cM.

All Al tolerance-associated DArT markers were assigned exclusively to rye chromosomes, and no association with the wheat genome was detected. A single marker that was highly associated with Al tolerance (wPt-3564: p-value E-09, *r^2 ^*= 0.22; see Table 3) and a less significant marker (rPt-401520, p = 0.0145) were mapped on 3R based on the triticale genetic map [19] as separated by about 130 cM (Figure 1). Among 4R markers (Figure 2), some associated and redundant markers mapped in proximity to one another. Two associated and four redundant markers assigned to the 4R chromosome mapped within a 3.8 cM region of the triticale genetic map. Some of these tightly linked markers were also identified on the rye genetic map [15], and they were within the same chromosomal region based on markers common to the two genetic maps (rye and triticale). Those markers exhibited the highest association values (E-07, *r^2 ^*= 0.146, see Table 3). Several other associated markers (E-04-E-06) were randomly distributed along the rye map and were missing in the triticale map. Two markers downstream of the linkage group represented by rPt-507784 and rPt-410768 also exhibited high association values (Table 3). Analysis of the 6R linkage maps showed the presence of three marker groups. One of them covered about 2.2 cM and consisted of three DArT markers with the highest p values (E-09 see Table 3) and *r^2 ^*about 0.17 (depending on the association method used) and was located on the triticale map, while two others (E-06 and E-05) were present on the rye map. Those two groups were separated by about 60 cM (Figure 3). One of the groups was approximately 10-20 cM from the group located on the rye map, while the other one was distal from it. Interestingly, the two linkage groups were not located in the same chromosomal region of the maps. DArT markers associated with Al tolerance and assigned to 7R formed a single linkage group on triticale [19] and another one on rye [15] genetic maps based on map alignment (Figure 4). The triticale linkage group consisted of two highly associated markers (E-08, *r^2 ^*= 0.186, see Table 3), while the one identified on the rye map was formed with less important markers (E-06, *r^2 ^*= 0.144). Many associated markers revealed via association mapping were not assigned to any of the linkage groups in the two maps.

### Mapping outliers

The majority of positive and balancing selection DArT markers were assigned to the chromosomes based on the known location of the markers. However, only some of them were located on the genetic maps of rye [15] and triticale [19].

A few markers related to positive selection were identified on the genetic maps. The rPt-508975 marker was present on the triticale, while rPt-390252 was detected on the rye genetic map [15] of the 3R chromosome (Figure 1). The two markers were separated from each other by more than 70 cM and were not in the vicinity of the associated markers. The markers rPt-400317 and rPt-505352 were located on the rye and triticale maps [19] on the 4R chromosome (Figure 2). These markers, as well as some balancing selection markers, were in close proximity to the cluster of associated markers (ca. 9 cM apart). The remaining outliers were slightly further apart from the associated marker. However, all of them formed a linked group that spread over approximately 20 cM, with outliers preferentially under positive selection located closer to the associated markers than those under balancing selection. There were few outliers in the 6R and none in the 7R linkage groups (Figure 3 and 4) that could be assigned to those chromosomes by means of available rye and/or triticale genetic maps.

## Discussion

Several factors need to be controlled when performing association mapping. First, the plant material must be properly selected and properly phenotyped. Then, after sample profiling with a carefully selected molecular marker system, redundant data and missing markers should be preferentially eliminated, genetic structure should be evaluated and statistical analyses can then be performed.

The plant material itself is possibly the most important factor [46,47]. For association studies, the most diverse or elite inbred lines [32,48,49], cultivars [33,50,51], and land races in the case of rice [52,53] should be used. Prior studies were based on 57 [54] to 577 [52] plants with the most common number ranging from 70 to 150 plants [26]. In this context, our mapping population consisting of 232 advanced breeding forms of triticale was quite large and above the lower limits used by others. Considering that triticale is predominantly a self-pollinated species and some of the forms that originated from double haploids were developed via anther culture, the plant material used is appropriate for association mapping studies with dominant marker platforms [14,55].

Another factor of importance for association studies is the careful phenotyping of plant materials to eliminate false associations between trait and markers [56]. In our experiments, we used a well described and widely explored test for Al tolerance [7]. Although the test is based on root regrowth, which is an indirect approach to the identification of tolerant plants, it is usually assumed to correlate well with the trait [57]. Nevertheless, it is obvious that Al tolerance is multigenic and a physiological test is not necessarily the best choice for screening plant material [58]. Another important issue is the use of relative rather than direct measures of a trait to enable comparison between different experiments [59]. The ratio between the root regrowth of a given plant and the longest root regrowth measured within the analyzed population is a possible measure. The ratios underwent arcsine transformation [59] and aluminum tolerance values were rescaled to new values to fulfill the statistical requirements of quantitative traits.

The DArT platform proved to be useful in association mapping performed on wheat [29,33], barley [34] and oat [31], and was a reasonable choice for our studies on advanced breeding forms of triticale, which identified several markers assigned to different chromosomes. However, it should be stressed that even using DArT markers it is practically impossible to avoid missing data that may appear at a level lower than 5% in the case of DArTs. Moreover, due to the high sensitivity of the approach, numerous rare markers with low frequencies were identified. Because such markers may influence linkage disequilibrium, they had to be removed from the analysis [60,61]. Similarly, identical markers or tightly linked ones (redundancy) may reduce the sensitivity of association mapping. Our approach nearly entirely eliminated or significantly reduced missing markers without involving mclust R-CRAN packages that are insensitive to such data [61]. Elimination of redundancy did not affect the information on markers that could be alternatives for the associated ones. Nevertheless, by using this approach, the advanced breeding forms used in each chromosomal set were reduced due to the merging of identical forms following the removal of missing and merging redundant markers. In some cases, the number of forms used dropped from 232 to 141. However, the chromosomal sets remained sufficiently large and exceeded the lower limits used by other studies [54].

The presence of population structure may result in spurious associations that could lead to numerous false positives [55,56]. To avoid such a problem, we used the agglomeration analysis implemented in the PAST software [62] and Bayesian statistics implemented in the Structure program [56]. Both methods indicated the presence of data structuring without separating winter and spring forms. Interestingly, while agglomeration and Bayesian approaches were capable of identifying data structuring, only the latter approach grouped individuals according to their known Al tolerance and was thus selected for routine use. Although calculations performed in the Structure software are time consuming due to the requirement for many burning periods, iterations and repetitions for each K value tested, they deliver information on the average genetic structure of the chromosomal sets required for association mapping in TASSEL [63], which is the most widely used software for association studies in plants [12,26,30,35]. It was used in similar studies on wheat and allowed for the identification of markers associated with traits such as response to stem rust, leaf rust, yellow rust, powdery mildew, grain field, heading date, flowering time, etc. [29,33].

Association mapping using both the GLM and MLM methods resulted in congruent results. However, the MLM approach usually provided higher *r^2 ^*values and a stronger association for the same markers than GLM. This confirms that the involvement of data structuring and relationships among analyzed forms improves the resolution of association mapping. An alternative approach based on Statistical Machine Learning (SML) to identify associated markers [64] was also applied. This approach has numerous advantages over the Bayesian method and it does not require time consuming analyses of population structure, as calculations are performed relatively fast [64]. It allowed identification of Al tolerance-associated markers that mostly, but not always, corresponded with those obtained in TASSEL. The discrepancies were possibly due to the fact that the whole data set rather than the chromosomal sets was used for calculations. Another possible explanation is the effect of structure on the results of association analysis. It will be interesting to compare the results with those of an SML algorithm that corrects for "structure", as this version has been developed recently (DArT PL, unpublished). In general, the smaller number of associated markers detected by SML is consistent with the more conservative (and likely more realistic) performance of this method when compared to several other techniques, as reported by Bedo et al. [64].

Based on known assignments of DArTs, Al-tolerant associated markers were localized to 3R, 4R, 6R and 7R chromosomes independently of the mapping approach used. No association was detected on the wheat genome. Our results are in agreement with several prior reports [40-42,65], indicating that Al-tolerant genes crucial for the expression of the trait in triticale are located on rye chromosomes rather than on wheat chromosomes. Considering that the strongest associations were in the 3R and 7R chromosomes, our results are congruent with those presented earlier [41,65,66] and our own results on several biparential triticale mapping populations (in preparation). Keeping in mind that most of our Al tolerance-associated markers have redundant counterparts, we succeeded in identifying as many as 52 candidate markers (46 via mapping in TASSEL and 14 following the SML approach, including eight markers identified via both methods).

Although association mapping may provide valuable information on associated markers and comparison with known DArTs may suggest their chromosomal assignments, their precise location is difficult to determine if saturated consensus genetic maps are not available. Unfortunately, such maps do not exist in triticale. However, a recently published report generated triticale and rye genetic maps using DArT markers [15,19]. Although rearrangements of rye chromosomes in the triticale genome in comparison to the rye genome may occur [67], changes in the distance between markers within several cM should not be a frequent occurrence. Thus, both maps could be used to verify whether associated markers assigned to the same chromosome fall within the same region or not. Such information proved to be valuable in estimating the putative location and the number of QTLs at least in the 4R and 6R chromosomes, where markers associated with the trait were located within a very short distance, which indicated the presence of two QTLs. Although few markers associated with Al tolerance were mapped to 3R, they were highly associated with the trait, indicating that a single QTL is present on the 3R chromosome. Similarly, at least two QTLs of different significance are present on 4R. The data for the 6R chromosome suggest that there might be as many as three QTLs, but only one seems to be highly significant. Finally, it is suggested that a single QTL represented by highly associated markers is also located on 7R, which is in agreement with previously published reports. Prior studies reported one QTL located on 3RS [41,42], 4RL [40,68] and 7RS [65]. Gallego and Benito [69] identified two isozyme loci linked to the rye Alt1 gene on chromosome 6R. This gene is probably the same as that located on chromosome arm 6RS (Alt1) by Anioł and Gustafson [40]. These results reported in the literature suggest that our QTLs may be located on the same arms of the chromosomes mentioned above. However, this localization is difficult to verify because prior studies used wheat-rye addition lines.

Genome regions under selection pressure for a given trait are likely to be involved in the expression of the trait [37]. Positive selection is considered to be responsible for adaptive traits [39]. The forms used in this study were selected for aluminum tolerance via many generative cycles; therefore, the identification of genomic regions under selection pressure (via markers called outliers), could be the method of choice to identify linked markers. Unfortunately, outliers reflecting genomic regions under positive selection located in the vicinity of markers highly associated with Al tolerance (ca 9 cM apart) were only present in the 4R linkage group. Moreover, some outliers under balancing selection were also within the same genomic region. Interestingly, all outliers indirectly mapped to the fourth chromosome covered the same region, extending over approximately 20 cM. In addition, the possibility that a single marker under positive selection could be close to the group of markers highly associated with the trait on 6R cannot be excluded. However, with the currently available rye and triticale maps (including possible discrepancies in synteny/colinearity between these genomes due to genome rearrangements), such a hypothesis is difficult to test. Similarly, another outlier under positive selection could be located in the proximity of the associated marker on 3R (ca 50 cM apart), while the other one did not appear to be linked to the second Al-associated marker. Our data confirm that outliers reflecting genomic regions under positive selection may be linked to the trait of interest, at least in the material used in this study. Nevertheless, it is evident that markers identified via analysis of outliers need independent confirmation of their value for MAS purposes.

## Conclusions

The DArT approach was used to generate numerous polymorphic markers for association mapping and to support the chromosomal location of the markers. Association mapping using GML, MLM and SML resulted in comparable results, although data obtained by SML differed to some extent from those derived by GLM and MLM. Involvement of genetic maps of rye and triticale allowed the grouping of markers according to their chromosomal positions and the identification of specific genomic regions (possibly QTLs) that could be involved in the expression of the trait. Outliers related to positive selection could be useful as additional candidate markers linked to the trait of interest.

## Methods

### Plant materials

The 232 triticale breeding forms used in the study were originated from the Experimental Station (Małyszyn, Poland) and consisted of 193 winter and 39 spring inbreed forms. Three winter triticale lines and 15 spring lines were double haploids (DH). Each triticale form was represented by a single, randomly selected plant.

### Al tolerance test

A standard Al tolerance physiological test was performed [7]. Triticale seeds were sterilized in 10% sodium hypochlorite for 10 minutes and then washed in water. After germination for 24 h at 10°C on moist filter paper in Petri dishes, they were transferred to a polyethylene net floated in a tray. The tray was filled with basic medium containing 2.0 mM CaCl_2_, 3.25 mM KNO_3_, 1.25 mM MgCl_2_, 0.5 mM (NH_4_)_2_SO_4 _and 0.2 mM NH_4_NO_3 _(pH 4.5), and left for 3 days under controlled-environment growth cabinet (POL-EKO-APARATURA, ST500 B40 FOT10) conditions at 25°C, photoperiod 12/12 h day/night, light intensity 40 W m^-2 ^and aeration. The plants were then transferred onto the same medium supplemented with AlCl_3 _(0.59 mM (16 ppm)). After 24 h, roots were washed with water and seedlings were placed again in the basic medium for 48 h. To assess tolerance levels, roots were stained in 0.1% Eriochrome Cyanine R for 10 minutes. The continued growth ability of roots was a measure of Al tolerance/sensitivity. To evaluate the response of seedling roots, the length of regrowth in mm was measured. The highly tolerant rye cultivars Dańkowskie Złote (winter rye) and Strzekęcińskie (spring rye) were used as controls.

### Phenotypic data transformation

The direct measures of root regrowth in mm were recalculated using the longest regrowth of all the seedlings as the denominator. Arcsine transformation was performed according to the formula arcsine square root (regrowth/the longest regrowth), where regrowth was measured in mm.

### DNA isolation

Total genomic DNA was isolated from fresh leaves of 14-day-old seedlings using the Plant DNeasy MiniKit 250 (Qiagen) following the manufacturer's instructions. DNA quantity was measured spectrophotometrically (NanoDrop ND-1000), and its integrity and purity was verified via electrophoresis on 1.2% agarose gels stained with EtBr (0.1 μg/ml) in TBE.

### Genotyping

The protocol for the AFLP fingerprinting followed that described by Vos et al. [70] with minor modifications according to Bednarek et al. [71]. Samples of genomic DNA (0.5 μg) were digested with *Eco*RI/*Mse*I, following ligation of adaptors and pre-selective amplification. For the selective amplification, we used eleven primer combination, E-ACA/M-CGC, E-ACC/M-CGG, E-ACG/M-CAC, E-ACG/M-CTG, E-ACG/M-CTC, E-ACT/M-CAC, E-AGC/M-CAG, E-AGC/M-CCG, E-ATC/M-CCA, E-ATG/M-CCC, E-AGT/M-CGT, where the E-XXX component was 32P-labeled. The products were separated on 7% polyacrylamide gels and visualized by autoradiography.

The following rye SSRs were assigned to the 7R chromosome and used under the experimental conditions and thermal profiles suggested by the owners of microsatellite bases: SCM 16, SCM 19, SCM 63, SCM 92, and SCM 150 (BAZ Database of *Secale cereale *Microsatellites, Federal Centre for Breeding Research on Cultivated Plants, Gros Lusewitz; [72]), and REMS 1162 and REMS 1188 (Rye Expressed Microsatellite Sites, [73]). Amplified products (PTC-225 Peltier Thermal Cycler (MJ Research)) were denatured and separated on a 7% denaturing polyacrylamide gel following overnight exposure to X-ray films at -35°C.

DArT marker analysis was performed by Diversity Arrays Technology P/L, Canberra, Australia using methods described by Tinker et al. [60].

### Data preparation for GLM and MLM

DArT molecular markers were transformed into binary (presence/absence) matrices and divided according to chromosome assignment. An additional matrix with unassigned markers (DArTs, SSRs and AFLPs) was also prepared.

When more than 30% of data were missing, individuals were removed from further analysis (seven forms out of 234). Each chromosomal marker set was checked for the presence of identical or similar plant forms using agglomeration analysis (UPGMA) and Dice genetic distance in PAST software [62]. The forms were assumed to be identical if the differences between them did not exceed 2% and if their molecular profiles, except when missing markers, were identical. The profiles of such individuals were merged, and missing markers were replaced by their counterparts from the other individual. The rationale for this was that even if two lines differed from each other (considering possible variation due to missing data), they were still significantly related and therefore representing them as a single entry would still be meaningful in association studies and should reduce redundancy. If a discrepancy in the Al tolerance of the individuals forming merged assemblies arose, then the highest value of the trait was assigned to the assembly.

Preliminary elimination of redundant markers was performed in the AFLPop ver. 1.1 excel add-in [74]. Due to numerous missing data, additional elimination steps were needed. Markers were clustered (UPGMA) using Dice genetic distance with PAST software, and those separated by a genetic distance lower than or equal to 2% (formed marker assembly) were merged. Missing data were completed using information from the redundant markers of the contiguous assembly. Only one representative of the given redundant marker assembly was retained, and information on the removed markers was saved for further analysis. Finally, low PIC markers, with a minor allele frequency of less than or equal to 5%, were also removed from analyses.

### Data structure

UPGMA clustering using Dice genetic distance was applied on the basis of the data from all non-redundant individuals and non-redundant markers of each chromosomal data set with PAST [62]. The robustness of the branches was estimated using 1000 bootstrap replicates.

Genetic structure was studied with STRUCTURE 2.2.3 program [75] following a Bayesian approach and using no admixture model or independent allele frequencies. Each simulation was run using burn-in and MCMC (Markov Chain Monte Carlo) lengths of 300 000. The range of possible Ks was tested from 1 to 10. Each simulation was run 10 times to quantify the amount of likely variation for each K. Estimation of the uppermost hierarchical level of the genetic structure was made using an *ad hoc *statistic ΔK and following the procedure described by Evanno et al. [76]. Computations were made using the BioPortal project [77].

### Average genetic structure

The average genetic structure of each chromosomal set was estimated in CLUMPP [78] based on ten Q matrices obtained in STRUCTURE for the given K.

### Linkage disequilibrium

For LD calculations, the correlation squared (*r*^2^) was used because it is relatively insensitive to small sample sizes and low allele frequencies [79]. Moreover, (*r*^2^) is adequate for mapping QTLs [26,79]. The General Linear Model (GML) and Multiple Linear Model (MLM) implemented in TASSEL software [63,80] were applied.

For the purpose of MLM analysis, kinship matrices adequate for dominant markers were evaluated in SPAGeDi [81]. Kinship matrix data concerning averaged structures were calculated using CLUMPP software and based on Q matrices.

### Statistical Machine Learning (SML)

Marker-trait associations were tested using SML technology as described by Bedo et al. [64]. The algorithms described in this paper were implemented as a "web service" by F. Detering (DArT PL, not published) on DArT PL's intranet. The software was run on the "non-redundant" marker set and a set of phenotypic data (see Phenotypic Data Transformation). For each marker in the dataset, the software calculates the PAVE value [64], which measures the contribution of this marker to the model by describing the phenotype as well as the probability (P) of this effect being observed by chance only. In addition, the software determines the complexity of the model (number of markers) contributing to phenotypic variation.

### Indirect location of markers on genetic maps

The information available on triticale [19] and rye [15] genetic maps saturated with DArTs was used to locate the markers indirectly on triticale chromosomes.

### Candidate genomic regions under selection pressure

Markers reflecting genomic regions under putative positive and balancing selection were identified by the Mcheza software [82]. The input data was organized based on the average structure of a chromosomal set obtained as described above. In an infinite allele model with 95000 simulations, "neutral" and "forced" mean F_*ST *_options were applied.

## Abbreviations

AFLP: Amplified Fragment Length Polymorphism; ALMT: Aluminum-Activated Malate Transporter; DArT: Diversity Arrays Technology; DH: Doubled Haploid; GLM: General Linear Model; LD: Linkage Disequilibrium; MAS: Marker Assisted Selection; MATE: Multidrug And Toxin Efflux; MLM: Multiple Linear Model; QTL: Quantitative Trait Locus/Loci; REMS: Rye Expressed Microsatellite Sites; SCM: *Secale cereale *Microsatellites; SML: Statistical Machine Learning; SSR: Simple Sequence Repeat; SSR: Single Sequence Repeats.

## Authors' contributions

AN carried out the physiological tests and molecular genetic studies, participated in running routine statistics, and wrote the manuscript. PTB conceived the study, participated in its design and coordination, performed part of the statistical analyses, and wrote the manuscript. GB and GC provided plant material. AK performed DArT analysis and SML statistics, and drafted the manuscript. AA provided intellectual input during the experiments and revised the manuscript. All authors read and approved the final manuscript.
